# Post-migration Social–Environmental Factors Associated with Mental Health Problems Among Asylum Seekers: A Systematic Review

**DOI:** 10.1007/s10903-020-01025-2

**Published:** 2020-05-19

**Authors:** Sohail Jannesari, Stephani Hatch, Matthew Prina, Sian Oram

**Affiliations:** 1 Health Service and Population Research, The Institute of Psychiatry, Psychology and Neuroscience, King’s College London, David Goldberg Building, 16 De Crespigny Park, London, SE5 8AF UK; 2 Psychological Medicine, The Institute of Psychiatry, Psychology and Neuroscience, King’s College London, London, UK; 3 ESRC Centre for Society and Mental Health, King’s College London, London, UK

**Keywords:** Refugees, Mental health, Asylum seeker, Discrimination

## Abstract

People seeking asylum are at an increased risk of mental disorder compared to refugees and other migrants. This paper aims to understand the impact of postmigration social–environmental factors to help inform efforts to reduce rates of mental disorder. We conducted a systematic review searching 11 databases, as well as 6 government and nongovernment websites. We asked 5 experts for recommendations, and carried out forwards and backwards citation tracking. From 7004 papers 21 were eligible and had the appropriate data. Narrative synthesis was conducted. 24 Social–environmental factors were identified and categorised into 7 themes: working conditions, social networks, economic class, living conditions, healthcare, community and identity, and the immigration system. Evidence suggests that discrimination and post-migration stress are associated with increased rates of mental disorder. The post-migration environment influences the mental health of people seeking asylum. Discrimination and post-migration stress are key factors, warranting further research and public attention.

**Electronic supplementary material:**

The online version of this article (10.1007/s10903-020-01025-2) contains supplementary material, which is available to authorized users.

## Introduction

The UN Refugee Agency [1] estimates there are 25.9 million refugees globally and 3.5 million people seeking asylum. Venezuela, Afghanistan, Syria, Iraq and the Democratic Republic of Congo constitute the top five nationalities for those seeking asylum [1]. In 2018, a decision was made on around a third of asylum applications (1.1 million) with about half of these (500,100) resulting in some form of humanitarian protection [1]. It is clear from these figures that the vast majority of applicants spend at least a year in the asylum system. During this time they must navigate an increasingly unwelcome sociopolitical atmosphere. Politicians such as US President Trump have presented people seeking sanctuary as a threat to national security [2]. The marginal EU public sympathy in 2015, when migration flows began to substantially increase, has crystallised into security and financial concerns (e.g. [3].).

Evidence suggests that people seeking asylum are at an increased risk of developing mental disorders compared to refugees and the host population [4]. Potential reasons can be located at different stages of migration: pre-migration, transit and post-migration [5, 6]. Mental health research usually focuses on pre-migration stressors, such as how traumatic experiences in countries of origin affect mental health in host countries [7, 8]. However, post-migration factors mediate the impact of pre-migration stressors on mental health [9–11]. Carswell et al. [12] and Gorst-Unsworth and Goldenberg [13] go further, suggesting that post-migration factors may be more important than pre-migration factors for some forced migrant populations. Gorst-Unsworth and Goldenberg found that only 11% of Iraqi refugees interviewed in the UK had PTSD though almost 65% had suffered physical torture in Iraq. Contrastingly, close to 44% had depression and this was primarily associated with a lack of social support in the UK.

In a brief literature review of post-migration risk factors related to asylum policy, Silove et al. [14] argued that low levels of financial support [13], the application process and harsh living conditions [15], as well as loneliness and boredom [16] were associated with as symptoms of depression, PTSD and anxiety. They did not include results from non-Western countries and the review is almost 20 years old. Patel [17] conducted a systematic review of English language papers, finding evidence that detention [18], length of the asylum process [19] and legal status [20] were associated with mental health outcomes such as PTSD symptoms, depression symptoms and psychopathology. Giacco’s recent review [21] included studies with both refugees and asylum seekers, suggesting that factors such as a sense of belonging (protective factor) and social isolation (risk factor) are associated with mental disorder. However, the review only considered papers from 2017 onwards and only 3 of the 29 eligible non-review studies included asylum seekers in their sample, with none solely working with asylum seekers.

This review focuses on social–environmental factors, defined by Barnett and Casper [22] as a person’s ‘immediate physical surroundings, social relationships, and cultural milieus’, including ‘built infrastructure; labour markets… power relations; government… [and] beliefs about place and community’. Social–environmental factors can change, either through medium-term individual actions or longer-term policy shifts. They are easier to adjust than most sociodemographic factors, character traits, and individual beliefs. Walsh et al. [23] evidence that positive psychology interventions based on character traits such can have poor acceptability and be perceived as belittling. Though skills and competencies such as language ability or vocational qualifications are changeable, they have been excluded to keep the review conceptually coherent and manageable.

An appreciation of social–environmental factors, such as the sociopolitical context in which forced migrants are received, can also lead to more ethical and effective mental health practices and interventions [24]. This is in line with Medical Research Council guidance [25] emphasising the importance of context, a term encompassing social–environmental factors, in the evaluation of complex interventions. Self-Help Plus, for instance, is a mental health intervention developed by the WHO for forced migrants in ‘low-resource humanitarian settings’ [26]. Accordingly, it was created with a consideration that many forced migrants live in environments with limited access to healthcare, and precarious legal and housing situations. Self-Help Plus also underwent a process of cultural adaptation [27], acknowledging the importance of social norms and beliefs in effective treatment.

This review, therefore, aims to identify, synthesise and appraise the evidence on the association of post-migration social–environmental factors with mental disorder in asylum seekers.

## Method

Systematic review, following the Preferred Reporting Items for Systematic Reviews and Meta-Analyses checklist and (a checklist is provided in Appendix A) registered with Prospero (CRD42017081915).

### Search Strategy

Online searches were in EMBASE, MEDLINE, Social Policy and Practice, PsychINFO, Web of Science, Dissertations and Global Theses, PTSD Publications, Cochrane Library, Cumulative Index to Nursing and Allied Health Literature, Latin American and Caribbean Health Sciences Literature and the Danish Institute Against Torture database. Grey literature databases OpenGrey and Global Health were also searched, as were the websites of several non-profit organisations (see Appendix B). Five experts provided suggestions on studies to include. We conducted forward and backward citation tracking for included studies after full-text screening.

Keyword and medical subject heading searches were conducted from 1 January 1967 to 19 July 2019. January 1967 was chosen as it was the date the UN Protocol Relating to the Status of Refugees was signed. This protocol extended refugee status to those affected by events outside of Europe. The search terms combined terms for mental disorders with terms for asylum seeking statuses [e.g. (exp PTSD/or “post-traumatic stress disorder” or PTSD) and (exp refugees/or asylum$ or refugee$ or migrant$)]. Full search terms are listed in Appendix B.

### Inclusion and Exclusion Criteria

Studies in any language working with asylum seekers over 18 years old were included. Studies with mixed samples where over 75% were asylum seekers were included. Research including only refugees or internally displaced people was excluded. Studies with populations living in detention, restricted reception centres and refugee camps were excluded. Such extreme living conditions may confound the relationship between asylum status and mental disorder, and are not representative of long-term post-migration living conditions. Studies also had to measure one or more mental disorders using either validated diagnostic or screening tools and to measure one or more social–environmental factor.

### Screening and Extraction

A two-stage screening process was used where title and abstract screening was followed by full-text screening. Two-hundred and fifty studies were independently subject to title and abstract screening by two researchers. There were 23 discrepancies (i.e. < 10%) which were discussed and resolved; the primary issue was uncertainty over whether a study included asylum seekers or refugees.

Information on study design, demographics, outcome measures, and results was extracted. Where data was missing or not disaggregated, authors were contacted. The Newcastle–Ottawa Assessment Scale [28] assessed quality for case–control and cohort studies, and an adapted version [29] was used for cross-sectional studies. We adapted the scale for use in the forced migration context (see Appendix C).

### Analysis

Analysis included description of study and population characteristics, calculation of odds ratios and 95% confidence intervals (if raw numbers were available). Narrative synthesis first sorted study variables into 24 common factors. Factors were then placed into conceptually coherent themes based on the International Organisation for Migration’s [30] social determinants of migrant health, an adaption of a World Health Organisation 2008 model [31]. Synthesis then followed the stages described by Popay et al. [32]: ‘developing a preliminary synthesis’—we organised the results in tables to identify patterns, ‘exploring relationships in the data’—we considered the role of study heterogeneity in emerging patterns, and ‘assessing the robustness of the synthesis product’—we evaluated the strength of evidence for each pattern. Synthesis was conducted for factors with six or more separate studies, and for studies reporting overall post-migration stress.

## Results

**Fig. 1:**
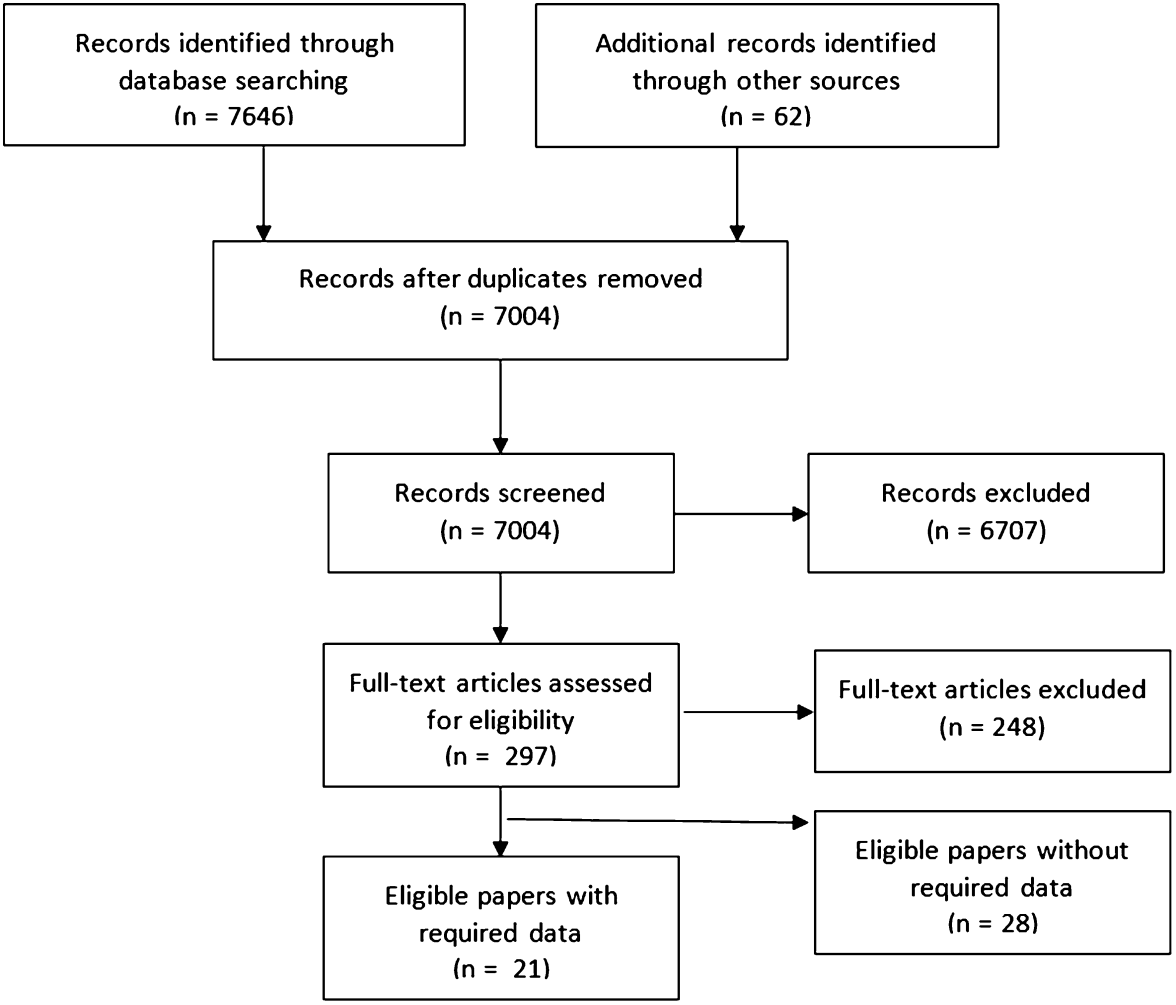
Study selection

We identified 7004 unique references (Fig. 1). After title and abstract screening, 297 papers remained for full-text screening, 49 of which were eligible (Appendix D). Of these, the required data could only be extracted from 11. After data requests, a further 10 studies were included to make 21 total.

**Fig. 2:**
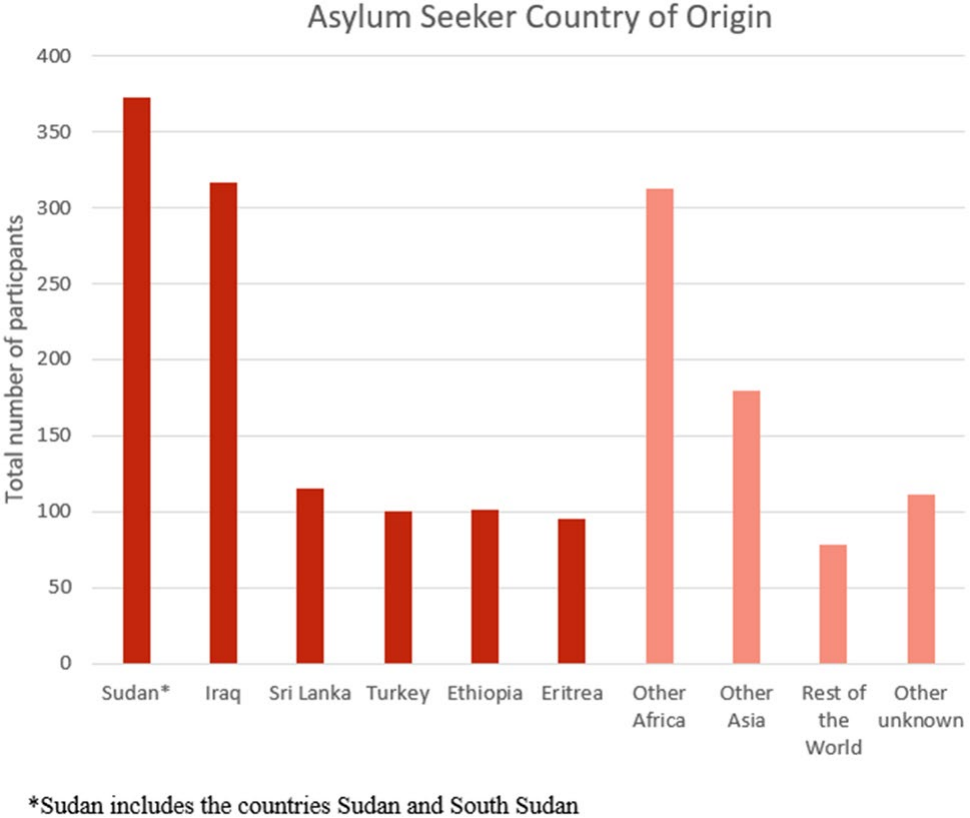
Top six asylum seeker countries of origin (n = 21 studies)

The number of adult asylum seekers in studies totalled 2402 (Table 1), with 1679 men and 856 women, and a median age of 34 (n = 15). Sudan and Iraq were the most common nationalities (Fig. 2). Research primarily occurred in high-income countries with majority white populations, with the USA (19% of studies) and Australia (19%) most frequent. 71% of studies were cross-sectional (29% prospective cohort) and 59% used convenience sampling. PTSD was the most commonly assessed outcome (90% of studies) followed by depression (86%) and anxiety (48%). Median prevalence for depression (n = 9) was 68% (IQR 50%, 85%), for anxiety (n = 6) 48% (IQR 46%, 61%), and PTSD (n = 10) 39% (IQR 36%, 51%).

**Table 1:** Study characteristics [15, 20, 33–51]

Lead author	Year	N	Gender		Age (x̄)	Countries of origin	Host	Design	Outcomes	Tool
			M	F						
Boersma	2005	117	70	47	41.6	Nigeria, Lebanon	USA	CS	Depression	HSCL-25
									Somatoform	SCL-90
Eisen	2016	78	33	45	34.1	Ethiopia, Cameroon	USA	Prospect. cohort	Depression	HSCL-25
									PTSD	HTQ-30
Hecker	2018	61	56	5	28.64	Afghanistan, Syria	Switzerland	CS	Depression	PHQ-9
									PTSD, CPTSD	ICD-11
Heeren	2012	86	60	16	29.8	African and the Middle East^a^	Switzerland	CS	Depression	MINI
									Anxiety, PTSD	
Hocking	2015	115	103	102	35.2	Sri Lanka	Australia	CS	Depression	HCSL-25
									Anxiety	HSCL-25
									PTSD	HTQ
Kaltenbach	2018	15	4	11	35.87	Syria, Iraq, Iran,	Germany	Prospect. cohort	Depression	PHQ-9
									PTSD	HTQ
Kashyap	2019	122	78	44	39.07	Ethnicities recorded	Australia	Prospect. cohort	Depression	PHQ-9
									PTSD	PCL
Laban	2005	294	190	104		Iraq	Netherlands	CS (Control)	Depression	CIDI
									Anxiety, PTSD, Somatoform	
Morgan	2017	42				African countries inc. Zimbabwe, DRC/Congo^a^	UK	CS	Depression	HCSL-25
									Anxiety	HSCL-25
									PTSD	HTQ
Müller	2018	78	33	45	38.2	Turkey	Germany	CS	Depression	ICD-10
									Anxiety, PTSD	
									Schizophrenia	
Nakash	2017	90	90	0	30.7	Sudan, Eritrean	Israel	CS	Depression	HSCL-25
									Anxiety	HSCL-25
									PTSD	PCL
Nickerson	2015	30	23	7		Turkey	Switzerland	CS	Depression	HSCL-25
									PTSD	PDS
Ryan	2008	162	202	152	32.5	Nigeria	Ireland	Prospect. cohort	Distress	SCL-90R
Schock	2015	50	30	20	32.1	Iran, Turkey, Balkans	Germany	Prospect. cohort	Depression	HCSL-25
									Anxiety	HCSL-25
									PTSD	PDS
Silove	1997	40	21	19	35	Data not available	Australia	CS	Depression	HCSL-25
									Anxiety	HCSL-25
									PTSD	CIDI
Slonim-Nevo	2015	340	276	64	30.6	Sudanese	Israel	CS	PTSD	PCL
Sohn	2019	129	93	36		Nigeria, Ethiopia	South Korea	CS	Depression	PHQ-9
									PTSD	IES-R
Song	2010	44	24	20	36	Iran, Eritrea, Iraq^a^	USA	CS	Depression	HSCL-25
									Anxiety	HSCL-25
									PTSD	PCL
Steel	1999	296	135	64	43.7	Sri Lanka	Australia	CS	PTSD	HTQ
Whitsett	2017	105	41	64	34.76	Ethiopia, Cameroon	USA	CS	Depression	HCSL-25
									Anxiety	HSCL-25
									PTSD	HTQ
Wong	2016	374	292	82	31.52	Not available	China-HK	CS	PTSD	PHQ-2

^a^Only main regions and countries displayed in table

**Fig. 3:**
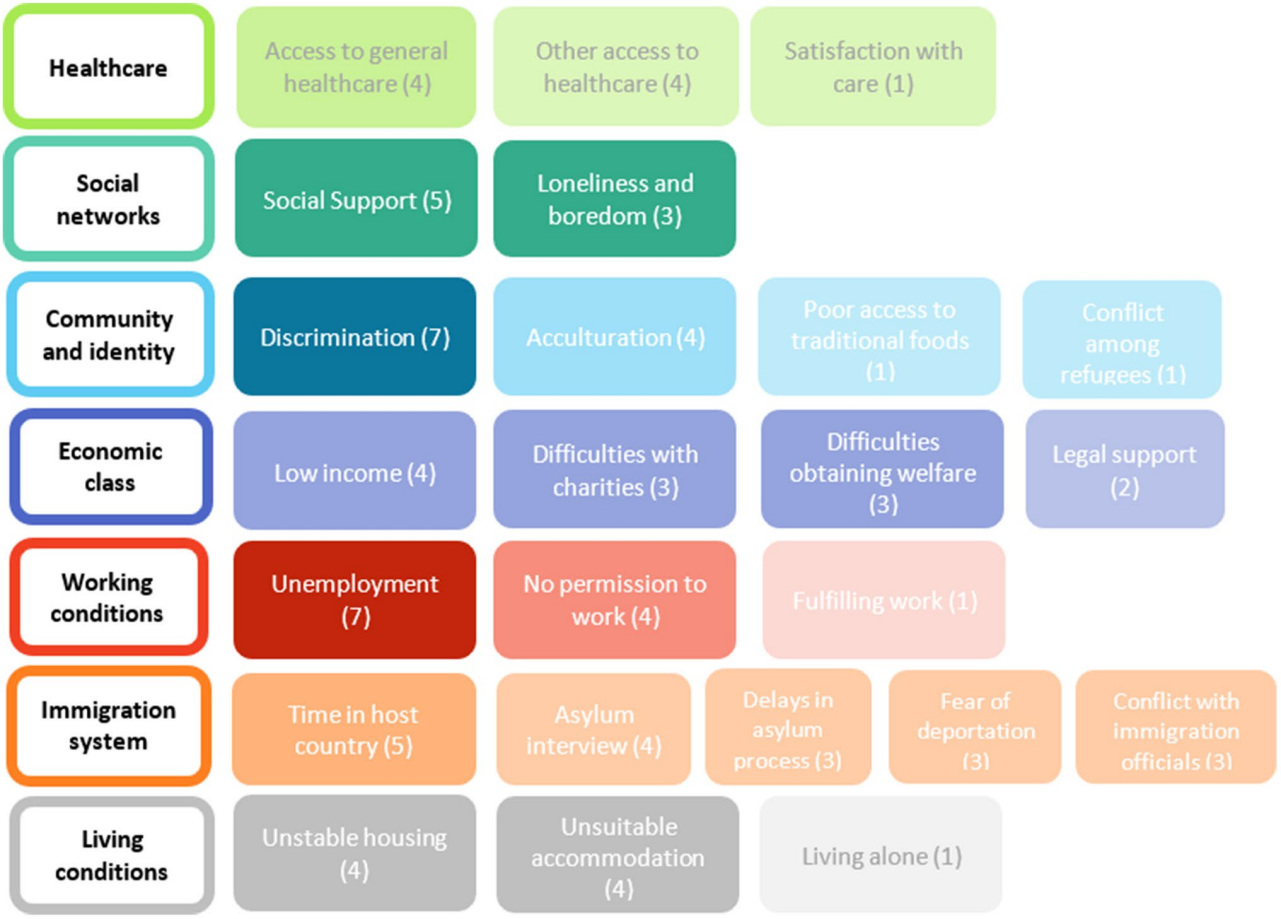
A map of social–environmental factors in included studies. Block shaded according to the number of studies; darker shading indicates more studies (number of studies in brackets)

Studies measured 24 social–environmental factors which we grouped into seven domains: working conditions, social networks, economic class, living conditions, healthcare, community and identity, and the immigration system (Fig. 3). The most frequently used risk factor tool was the Post-Migration Living Difficulties Questionnaire (PMLD, 10 studies), developed by Silove et al. [15].

Immigration system was the most examined category (18 studies), including the largest variety of factor types (5). Social networks received the least attention (8 studies) and had the fewest factor types (2). Although all studies focussed on mental health, access to counselling was only reported by two studies [15, 41]. Items in this theme were limited, seldom considering the impact of culture and language on healthcare quality.

Factors were typically broadly defined. In examining the asylum interview, several papers [15, 20, 49] asked whether ‘interviews by immigration officials’ were a source of stress. However, this could include immigration officers at the border, interviewers in an asylum interview or tribunal judges in court. Schock et al. [45] provided the exception, focussing on the asylum interview, breaking it down different potential sources of stress: ‘perceived justice of the hearing’, ‘testimony stress’ and ‘delay stress’. Similarly, some factors combined potentially separate risk factors into one. Loneliness and boredom was created as a factor group because studies often grouped them [20, 35, 41].

Three studies [40, 44, 51] provided strong evidence that discrimination is associated with higher rates of mental disorder and four [15, 20, 35, 41] found weak evidence of an association. The former set had larger samples and used several questions to arrive at a discrimination score, contrasting with the single item discrimination statement used in the other studies.

In their study with 294 Iraqi asylum seekers, Laban et al. [40] found a strong association between increased levels of discrimination and increased depression, anxiety and somatoform disorder (p =  < 0.01 for all results). In their multivariate analysis, Wong et al. [51] found a small association between everyday discrimination and depression (OR = 1.2, 95% CI 1.10–1.24) among a sample of 374 African asylum seekers in Hong Kong. Ryan et al. [44] worked with 162 people from 38 different countries, finding that discrimination was positively associated with distress in their multiple regression (β = 0.29, p < 0.001).

The four other studies generally found no association between discrimination and mental disorders in their analyses. Silove et al. [15] did, however, find an association between discrimination and increased PTSD (95% CI 4.52–22.50), Morgan et al. [41] with decreased anxiety (r =  − 0.36, p = 0.02), Nickerson et al. [20] with increased depression (OR = 5, 95% CI 4.54–49.44) and Hecker et al. [35] with complex PTSD (OR = 3.6, 95% CI 3.16–17.6). Confidence intervals in all studies were broad due to small sample sizes, while results were not part of a multivariate model accounting for confounders.

Two studies [37, 43] found evidence for an association between unemployment and mental disorder, with five [33, 34, 39, 41, 47] finding no evidence. In their study with 90 asylum seekers in Israel, Nakash et al. [43] found that unemployment was associated with higher rates of depression (OR = 2.1, 95% CI 1.04–5.09). Hocking et al. [37] also found an association between depression and unemployment (OR = 2.16, 95% CI 1.02–4.56) in their study with 115 people seeking asylum in Australia.

Morgan et al. [41] found no evidence of a relationship between ‘not being able to find work’, a proxy for unemployment, and anxiety (r = 0.21, p = 0.182), depression (r = 0.035, p = 0.826) or PTSD (r =  − 0.106, p = 0.504). Eisen [34] found no evidence of an association between unemployment and PTSD (β =  − 0.029, p = 0.766) or depression (β =  − 0.036; p = 0.712), a pattern repeated for Sohn [47] with depression (OR = 1.19, 95% CI 0.21–6.61) and PTSD (OR = 1.821, 95% CI 0.34–9.91), Kashyap et al. [39] for depression (β =  − 0.1; p = 0.28) and PTSD (β =  − 0.06; p = 0.51), and Boersma [33] for depression (r =  − 0.033, p = 3.61) and somatization (r =  − 0.04, p =  − 0.336). Results could be confounded because participants working without permission do not want to reveal this to researchers. Only Eisen [34] considered work authorisation, using an employment rating scale developed by the Advocates for Survivors of Torture and Trauma charity (cited in Eisen, p. 41).

Five studies [20, 40, 42, 44, 49] reported a score for general post-migration living difficulties derived from some of the factors in Fig. 3. Four of these studies reported that post-migration problems are associated with increased odds of mental disorder—all aside from Muller et al. [42]. The majority (4 of 5 studies) used a measure derived from the 23-item PMLD developed by Silove et al. [15]. Both Ryan et al. [44] and Nickerson et al. [20] used a condensed form of the checklist tailored to their study context (17 and 13 items respectively).

Nickerson et al. [20] found that increases in migration living difficulties were associated with higher rates of depression (total effects^1^ = 0.06, p =  < 0.001) and PTSD (total effects = 0.07, p =  < 0.001). Ryan et al. [44] similarly found that higher overall scores on their post-migration checklist was related to higher rates of distress (β = 0.44, p =  < 0.000). Though Muller et al. [42] did not conduct any statistical comparison, there appeared to be no difference in the number of migration-related stressors between Turkish people seeking asylum in Germany with PTSD and those without (5.25 stressors against 6, in a sample of 16 and 13 respectively).

Ryan et al.’s [44] principal component analysis (PCA) identified three groups of post-migration living difficulties; higher scores in each were associated with increased rates of distress: basic living difficulties (r = 0.56 p = 0.000), asylum stress (ρ = 0.27 p = 0.001) and family separation (r = 0.02, p = 0.005). Laban et al.’s [40] factor analysis created the similar categories: family issues, the asylum procedure, socioeconomic living conditions and discrimination, and socioreligious living conditions. Increases in category scores associated with increases anxiety, depression and somatoform disorder (p < 0.05 for all categories). Steel et al.’s [49] PCA produced the themes: residency determination; threat to family; health care, welfare and asylum; adaptation difficulties and loss of culture and support. Results from the latter three were reported and higher scores were positively associated with posttraumatic symptoms (β = 0.24, 0.33 and 0.27 respectively).

The categories developed through PCA and factor analysis were not always conceptually coherent. In the paper from Steel et al. [49] ‘healthcare, welfare and asylum’ included elements as disparate as ‘poor access to emergency medical care’, ‘delays in processing your application’ and ‘little help with welfare from charities’. In Ryan et al.’s [44] paper ‘basic living difficulties’ encompassed ‘racism and discrimination’, ‘financial concerns’ and ‘dietary concerns’, and there was overlap with the ‘asylum stress’ category which included ‘work permission’.

## Discussion

Our review identified 24 social–environmental risk factors for asylum seeker mental health from 21 papers and categorised them into 7 domains: working conditions, social networks, economic class, living conditions, healthcare, community and identity, and the immigration system. Most risk factors were examined by only a few studies, but synthesis of findings was possible for discrimination (community and identity), unemployment (working conditions), and post-migration stress (encompassing multiple domains). Strengths of the review included its comprehensive approach and robust search strategy. However, despite requests to corresponding authors for assistance, data could not be extracted for a large proportion of eligible papers. Consequently, work from research hubs in Australia and the Netherlands was excluded, and significant nationality groups such as Syrians and Afghans underrepresented.

Findings suggested a link between discrimination and mental disorder, though study setting and measurement approaches were heterogeneous. The larger studies, using more nuanced scales to investigate different facets of discrimination, more consistently reported an association between discrimination and mental disorder. In comparison, the smaller studies used single-item measures. The seven studies examining this relationship were conducted in different countries and, largely, with different nationalities. Experience of discrimination may vary by setting and by asylum seeker nationality and ethnicity [52], it is not clear whether its impact on mental disorder also varies. However, our findings reflect the broader literature on mental health and discrimination, including from large meta-analyses [53, 54] as well as findings from studies conducted with refugees [55].

In contrast with the wider mental health literature [17], findings from the seven studies assessing unemployment suggested only a weak positive association with mental disorder. However, the majority of studies investigating this association did not consider a potential confounder: working without authorisation, which may be common but not readily disclosed [56]. Those working without permission may be subject to additional stressors such as forced labour, unpaid wages and a lack of institutional recourse [57]. Future studies could use scales that incorporate unauthorised working, such as the employment scale used by Eisen et al. [34].

There was good evidence suggesting that post-migration stress as a broad category is associated with higher rates of mental disorder, with four of the five studies investigating this finding evidence for an association. Our work reinforces recent appeals from academics to consider post-migration factors in greater depth [58]. Further research could explore domains within post-migration stress, such as living conditions and healthcare in more detail. Factors relating to the asylum process were key components of all of general post-migration stress score measures. As a category readily changeable through government policy, this could form a focus for future research and advocacy. The broad cross-cutting categories around post-migration developed through PCA were not always conceptually coherent. The categories developed in this review could provide a basis for grouping factors in future studies.

The included studies examined a broad range of risk factors, exemplified by the reliance on the Post-Migration Living Difficulties checklist which uses single item questions for complex issues such as discrimination. Even in the domain with the greatest number of individual risk factors (the immigration system) risk factors were wide-ranging and ill-defined. Schock et al. [45] provide a useful model for future research by focussing on the substantive asylum interview and breaking down different potential sources of stress. Similarly, measurement of access to healthcare could have been improved by recognising the impact of culture and language on the quality of healthcare. Nellums et al. [59] have found, for example, that healthcare access for people seeking asylum was inhibited by the lack of translators or the use of inappropriate translators (friends and family, or male interpreters for women’s sexual health services).

Understanding of how post-migration environmental factors impact the mental health of people seeking asylum would be deepened if studies asked separate questions about related but distinct concepts. For example, while Morgan et al. [41] asked participants about ‘mistakes and delays in the application process’, Jannesari et al. [60] have reported that people seeking asylum have different experiences and reactions to delays as opposed to mistakes (particularly trivial ones with significant consequences). Similarly, while many studies combined loneliness and boredom, Ryan et al.’s [44] Post-Arrival Concern Checklist assessed them as separate concepts.

Our comprehensive search strategy, accepting papers from all languages, looking at any mental disorder and social–environmental risk factor, is a key strength of our review. As is the large range of databases and sources used to identify papers. A major limitation was the number of eligible papers for which data could not be extracted (28 of 49).

## Conclusion

It has long been established that people seeking asylum have a high prevalence of mental disorder compared to host and other migrant populations. This review begins to address why this might be. Findings suggest both discrimination and general post-migration stress are linked to an increased risk of mental disorder, with a possible protective role for employment. With anti-migrant rhetoric increasing in many places worldwide, discrimination and restrictions on the freedoms and entitlements of people seeking asylum could increase, with potentially negative consequences for mental health.

### New Contribution to the Literature

This review considers why the prevalence of mental disorder is high among people seeking asylum compared to other migrants and refugees. It investigates the role of social–environmental risk factors in the post-migration period. No limits were placed on language, country, or type of mental disorder. We synthesise evidence on a vast array of risk factors (often using data initially unreported in papers) into seven risk domains: working conditions, social networks, economic class, living conditions, healthcare, community and identity, and the immigration system. Findings suggest that discrimination and post-migration stress are associated with an increased risk of mental disorder in people seeking asylum.

With anti-migrant rhetoric increasing in many places worldwide, discrimination against people seeking asylum could increase, with potentially negative consequences for mental health. The reduction of discrimination should be considered when forming asylum policy. Several risk domains, including living conditions and healthcare, remain under-researched and should be explored in future work. We also recommend the use of more nuanced measures of discrimination, employment and other risk factors to arrive at more meaningful findings.

## Electronic supplementary material

Below is the link to the electronic supplementary material.

Supplementary file1 (DOCX 52 kb)

## Footnotes

1 ‘Total effects are the sum of direct and indirect effects that connect a predictor to an outcome’ [61].
