# Supplementary material for: Post-migration Social–Environmental Factors Associated with Mental Health Problems Among Asylum Seekers: A Systematic Review
Source: J Immigr Minor Health. 2020 May 19;22(5):1055–64. doi: 10.1007/s10903-020-01025-2 (PMC7441054; doi:10.1007/s10903-020-01025-2)
Supplement: Supplementary file 1 — Supplementary file1 (DOCX 52 kb) [file 10903_2020_1025_MOESM1_ESM.docx]

**Appendix A**

| **Section/topic** | **#** | **Checklist item** | **Reported on page #** |
| --- | --- | --- | --- |
| **TITLE** | | |  |
| Title | 1 | Identify the report as a systematic review, meta-analysis, or both. | 2 |
| **ABSTRACT** | | |  |
| Structured summary | 2 | Provide a structured summary including, as applicable: background; objectives; data sources; study eligibility criteria, participants, and interventions; study appraisal and synthesis methods; results; limitations; conclusions and implications of key findings; systematic review registration number. | Abstract |
| **INTRODUCTION** | | |  |
| Rationale | 3 | Describe the rationale for the review in the context of what is already known. | 1-2 |
| Objectives | 4 | Provide an explicit statement of questions being addressed with reference to participants, interventions, comparisons, outcomes, and study design (PICOS). | 2 |
| **METHODS** | | |  |
| Protocol and registration | 5 | Indicate if a review protocol exists, if and where it can be accessed (e.g., Web address), and, if available, provide registration information including registration number. | 2 |
| Eligibility criteria | 6 | Specify study characteristics (e.g., PICOS, length of follow-up) and report characteristics (e.g., years considered, language, publication status) used as criteria for eligibility, giving rationale. | 3 |
| Information sources | 7 | Describe all information sources (e.g., databases with dates of coverage, contact with study authors to identify additional studies) in the search and date last searched. | 2-3 |
| Search | 8 | Present full electronic search strategy for at least one database, including any limits used, such that it could be repeated. | Appendix B |
| Study selection | 9 | State the process for selecting studies (i.e., screening, eligibility, included in systematic review, and, if applicable, included in the meta-analysis). | 3 |
| Data collection process | 10 | Describe method of data extraction from reports (e.g., piloted forms, independently, in duplicate) and any processes for obtaining and confirming data from investigators. | 3-4 |
| Data items | 11 | List and define all variables for which data were sought (e.g., PICOS, funding sources) and any assumptions and simplifications made. | 3 |
| Risk of bias in individual studies | 12 | Describe methods used for assessing risk of bias of individual studies (including specification of whether this was done at the study or outcome level), and how this information is to be used in any data synthesis. | 3-4 |
| Summary measures | 13 | State the principal summary measures (e.g., risk ratio, difference in means). | 3 |
| Synthesis of results | 14 | Describe the methods of handling data and combining results of studies, if done, including measures of consistency (e.g., I^2^) for each meta-analysis. | 4 |
| Risk of bias across studies | 15 | Specify any assessment of risk of bias that may affect the cumulative evidence (e.g., publication bias, selective reporting within studies). |  |
| Additional analyses | 16 | Describe methods of additional analyses (e.g., sensitivity or subgroup analyses, meta-regression), if done, indicating which were pre-specified. |  |
| **RESULTS** |  |  |  |
| Study selection | 17 | Give numbers of studies screened, assessed for eligibility, and included in the review, with reasons for exclusions at each stage, ideally with a flow diagram. | 4-5 |
| Study characteristics | 18 | For each study, present characteristics for which data were extracted (e.g., study size, PICOS, follow-up period) and provide the citations. | 6-7 |
| Risk of bias within studies | 19 | Present data on risk of bias of each study and, if available, any outcome level assessment (see item 12). | 7-12 |
| Results of individual studies | 20 | For all outcomes considered (benefits or harms), present, for each study: (a) simple summary data for each intervention group (b) effect estimates and confidence intervals, ideally with a forest plot. | 7-12 |
| Synthesis of results | 21 | Present results of each meta-analysis done, including confidence intervals and measures of consistency. |  |
| Risk of bias across studies | 22 | Present results of any assessment of risk of bias across studies (see Item 15). |  |
| Additional analysis | 23 | Give results of additional analyses, if done (e.g., sensitivity or subgroup analyses, meta-regression [see Item 16]). |  |
| **DISCUSSION** |  |  |  |
| Summary of evidence | 24 | Summarize the main findings including the strength of evidence for each main outcome; consider their relevance to key groups (e.g., healthcare providers, users, and policy makers). | 12-15 |
| Limitations | 25 | Discuss limitations at study and outcome level (e.g., risk of bias), and at review-level (e.g., incomplete retrieval of identified research, reporting bias). | 15 |
| Conclusions | 26 | Provide a general interpretation of the results in the context of other evidence, and implications for future research. | 12-15 |
| **FUNDING** |  |  |  |
| Funding | 27 | Describe sources of funding for the systematic review and other support (e.g., supply of data); role of funders for the systematic review. | 16 |
| Risk of bias across studies | 15 | Specify any assessment of risk of bias that may affect the cumulative evidence (e.g., publication bias, selective reporting within studies). |  |

*From:*  Moher D, Liberati A, Tetzlaff J, Altman DG, The PRISMA Group (2009). Preferred Reporting Items for Systematic Reviews and Meta-Analyses: The PRISMA Statement. PLoS Med 6(7): e1000097. doi:10.1371/journal.pmed1000097

**Appendix B**

**Ovid Search Strategy (Embase (including classic), Medline (including ahead of print), Social Policy and Practice, Psychinfo, Global Health)**

1. "Depressive Disorder"/co, di, dh, dt, ec, ep, eh, et, hi, mo, nu, pc, px, rh, sn, su, th

2. "anxiety disorders"/co, di, dh, dt, ec, ep, eh, et, hi, mo, nu, pc, px, rh, sn, su, th

3. ((combat or neurotic or reactive or somatoform or somati$ation or adjustment or dissociat$) adj disorder$).mp.

4. PTSD/co, di, dh, dt, ec, ep, eh, et, hi, mo, nu, pc, px, rh, sn, su, th

5. schizophrenia/co, di, dh, dt, ec, ep, eh, et, hi, mo, nu, pc, px, rh, sn, su, th

6. (psychotic or suicidality or "delusional disorder").mp.

7. ((“substance abuse” or “drug abuse”) adj500 mental).mp. 🡪 could put and here?

8. "personality disorder"/co, di, dh, dt, ec, ep, eh, et, hi, mo, nu, pc, px, rh, sn, su, th

9. ("avoidant personality disorder" or "narcissistic personality disorder" or " obsessive compulsive personality disorder" or " anankastic personality disorder" or psychopath or psychopathy or psychopathic).mp.

10. eating disorders/co, di, dh, dt, ec, ep, eh, et, hi, mo, nu, pc, px, rh, sn, su, th

11. (compulsive adj (eat$ or vomit$ or purg$)).mp.

12. bipolar disorder/co, di, dh, dt, ec, ep, eh, et, hi, mo, nu, pc, px, rh, sn, su, th

13. (neurosis or psychoneurosis).mp.

14. (asylum seek$ or refugee$ or migrant$ or immigrant$ or "forcibly displaced").ti.

15. refugees/

16. 14 or 15

17. 1 or 2 or 3 or 4 or 5 or 6 or 7 or 8 or 9 or 10 or 11 or 12 or 13

18. (16 and 17) not neuroscience$.mp. not [neurology.mp](http://neurology.mp/). not neurochem$.mp. not neurotransmitter$.mp. not neuropsy$.mp. not immun$.mp. not vitamin$.mp.

19. (18 and ((child$ or adolescent or teenager or pubescent).mp. or child/) and (adult/ or (aged or elderly or pensioner).mp.)) or (18 not ((child$ or adolescent or teenager or pubescent).mp. or child/))

20. (19 and ("second generation" or "second-generation" or "2nd generation").mp. and ("first-generation" or "first generation" or "1st generation").mp.) or (19 not ("second generation" or "second-generation" or "2nd generation").mp.)

21. Limit 20 to (humans and yr="1967 -Current")

**Web of Science Search Strategy (Science Expanded, SSCI)**

#1 ((((((((TS=("Depressive Disorder" or depression or dysthymic disorder) OR TS=("anxiety disorder" or "panic disorder" or anxiety or agoraphobia or separation anxiety or neurosis or psychoneurosis) OR TS=("obsessive-compulsive" or "obsessive compulsive") OR TS=(PTSD or "post-traumatic stress disorder" or "post traumatic stress disorder" or "posttraumatic stress disorder") OR TS=((combat or neurotic or reactive or somatoform or somati$ation or adjustment or dissociat*) near/1 disorder$) OR TS=(schizophrenia or psychotic or suicidality or "delusional disorder") OR TS=("substance abuse" or "drug abuse") OR TS=("personality disorder" or "avoidant personality disorder" or "narcissistic personality disorder" or " obsessive compulsive personality disorder" or "anankastic personality disorder" or psychopath or psychopathy or "antisocial personality disorder" or "borderline personality disorder" or "compulsive personality disorder" or "dependent personality disorder" or "histrionic personality disorder" or "paranoid personality disorder" or "passive-aggressive personality disorder" or "schizoid personality disorder" or "schizotypal personality disorder" or psychopathic) OR TS=("eating disorders" or "Anorexia Nervosa" or "Binge-Eating Disorder" or "binge eating disorder" or "Bulimia Nervosa" or ((eat* or vomit* or purg*) near/1 compulsive)) OR TS=("bipolar disorder" or mania or manic))

AND TI=(asylum seeker$ or refugee$ or migrant* or immigrant* or "forcibly displaced"))

NOT TS=(neuroscience$ or neurology or neurochem* or neurotransmitter$ or neuropsy* or immun$ or vitamin$))))))))

#2 TS=(child* or adolescent or teenager or pubescent)

#3 #2 AND #1 AND TS=adult

#4 #1 NOT TS=(child* or adolescent or teenager or pubescent)

#5 #3 OR #4

#6 TS=("second generation" or "second-generation" or "2nd generation")

#7 #6 AND #5 AND TS=("first-generation" or "first generation" or "1st generation")

#8 #5 NOT TS=("second generation" or "second-generation" or "2nd generation")

#9 #7 OR #8

#10 (#9 NOT ((((WC=(law or toxicology or medicine research experimental or agriculture dairy animal science or pharmacology pharmacy or cell biology or chemistry medicinal or education scientific disciplines or engineering biomedical or oncology or pediatrics or anesthesiology or endocrinology metabolism or genetics heredity or history philosophy of science or industrial relations labor or infectious diseases or evolutionary biology or international relations or orthopedics or management or marine freshwater biology or dentistry oral surgery medicine or metallurgy metallurgical engineering or microbiology or obstetrics gynecology or respiratory system or mining mineral processing or ophthalmology or cardiac cardiovascular systems or planning development or gastroenterology hepatology or plant sciences or immunology )))

NOT SU=(biodiversity conservation or science technology other topics or integrative complementary medicine or legal medicine or neurosciences neurology or pathology or engineering or transportation or urology nephrology )))

AND document types: (article)indexes=sci-expanded timespan=1967-2018

**Proquest (PTSD Publications, Dissertation and Global Theses)**

(((((MESH("Antisocial Personality Disorder") OR MESH("Mental Health") OR MESH("Mental Disorder") OR MESH(Depression) OR MESH("Post-Partum Depression") OR MESH("Major Depressive Disorder") OR MESH("Dysthymic Disorder") OR MESH(anxiety) OR MESH("Eating Disorders") OR MESH("Histrionic Personality Disorder") OR MESH("Anxiety Disorders") OR MESH("Borderline Personality Disorder") OR MESH("Personality Disorders") OR MESH("post-traumatic stress disorders") OR MESH("Depressive Disorders") OR MESH("Paranoid Personality Disorder") OR MESH("Dependent Personality Disorder") OR MESH("Bipolar Disorders") OR MESH("Passive-Aggressive Personality Disorder") OR MESH("Obsessive-Compulsive Personality Disorder") OR MESH("Schizophrenia"))) OR ((combat OR neurotic OR reactive OR somatology OR somati?ation OR adjustment OR dissociation OR dissociat*) NEAR/1 disorder*) OR (psychotic OR suicidality OR "delusional disorder" OR psychosis) OR (("substance abuse" OR "drug abuse") NEAR/500 mental) OR (anxiety OR "panic disorder") OR ("post-traumatic stress disorder" OR ptsd OR "post traumatic stress disorder" OR "posttraumatic stress disorder") OR ("common mental disorder" OR "common mental illness" OR "psychiatric disorder" OR "psychiatric disease") OR (((depression OR dysthymic) NEAR/500 mental) OR ((depression OR dysthymic) NEAR/500 disorder))) AND ((MESH("Refugees")) OR TI(asylum seek* OR refugee? OR migrant? OR immigrant? OR "forcibly displaced" OR "war survivor" OR "uprooted person"))) NOT (Neuroscience*. OR neurology* OR neurochem* OR neurotransmitter* OR neuropsy* OR immun* OR vitamin*)) AND pd(20180301-20190801)

**EBSCO (CINAHL)**

((MH("Antisocial Personality Disorder") OR MH("Eating Disorders") OR MH("Histrionic Personality Disorder") OR MH("Anxiety Disorders") OR MH("Borderline Personality Disorder") OR MH("Personality Disorders") OR MH("post-traumatic stress disorders") OR MH("Depressive Disorders") OR MH("Paranoid Personality Disorder") OR MH("Dependent Personality Disorder") OR MH("Bipolar Disorders") OR MH("Passive-Aggressive Personality Disorder") OR MH("Obsessive-Compulsive Personality Disorder") OR MH("Schizophrenia")

OR

(AB((combat OR neurotic OR reactive OR somatology OR somati?ation OR adjustment OR dissociation or dissociat*) NEAR/1 disorder*) OR (psychotic or suicidality or "delusional disorder") OR ((“substance abuse” or “drug abuse”) near/500 mental) OR ("avoidant personality disorder" OR "narcissistic personality disorder") OR ("obsessive compulsive personality disorder" OR " anankastic personality disorder" OR psychopath*) OR (compulsive NEAR/1 (eat* OR vomit* OR purg*)) OR (neurosis OR psychoneurosis))

NOT

(Neuroscience*. or neurology* or neurochem* or neurotransmitter* or neuropsy* or immun* or vitamin*)))

AND

(MH(refugees) or AB(asylum seek* or refugee* or migrant* or immigrant* or "forcibly displaced"))

**Cochrane Library**

#1 MeSH descriptor: [Depressive Disorder] this term only

#2 MeSH descriptor: [Anxiety Disorders] this term only

#3 MeSH descriptor: [Stress Disorders, Post-Traumatic] this term only

#4 MeSH descriptor: [Schizophrenia] this term only

#5 MeSH descriptor: [Feeding and Eating Disorders] this term only

#6 MeSH descriptor: [Bipolar Disorder] this term only

#7 ((combat or neurotic or reactive or somatoform or somati$ation or adjustment or dissociat$) near/1 disorder$)

#8 psychotic or suicidality or "delusional disorder"

#9 (("substance abuse" or "drug abuse") and mental)

#10 ("avoidant personality disorder" or "narcissistic personality disorder" or "obsessive compulsive personality disorder" or "anankastic personality disorder" or psychopath or psychopathy or psychopathic)

#11 (compulsive adj(eat$ or vomit$ or purg$))

#12 (neurosis or psychoneurosis)

#13 (asylum seek$ or refugee$ or migrant$ or immigrant$ or "forcibly displaced")

#14 MeSH descriptor: [Refugees] this term only

#15 #13 or #14

#16 #1 or #2 or #3 or #4 or #5 or #6 or #7 or #8 or #9 or #10 or #11 or #12

#17 #15 and #16

#18 #17 not (neuroscience$ or neurology or neurochem* or neurotransmitter$ or neuropsy* or immun$ or vitamin$)

**VHL Regional Portal (LILACS)**

(("Depressive Disorder" or depression or dysthymic disorder) OR ("anxiety disorder" or "panic disorder" or anxiety or agoraphobia or separation anxiety or neurosis or psychoneurosis) OR ("obsessive-compulsive" or "obsessive compulsive") OR (PTSD or "post-traumatic stress disorder" or "post traumatic stress disorder" or "posttraumatic stress disorder") OR (“combat disorder” or “neurotic disorder” or “reactive disorder” or “somatoform disorder” or “somati$ation disorder” or “adjustment disorder” or “dissociation disorder”) OR (schizophrenia or psychotic or suicidality or "delusional disorder") OR ("substance abuse" or "drug abuse") OR ("personality disorder" or "avoidant personality disorder" or "narcissistic personality disorder" or " obsessive compulsive personality disorder" or "anankastic personality disorder" or psychopath or psychopathy or "antisocial personality disorder" or "borderline personality disorder" or "compulsive personality disorder" or "dependent personality disorder" or "histrionic personality disorder" or "paranoid personality disorder" or "passive-aggressive personality disorder" or "schizoid personality disorder" or "schizotypal personality disorder" or psychopathic) OR ("eating disorders" or "Anorexia Nervosa" or "Binge-Eating Disorder" or "binge eating disorder" or "Bulimia Nervosa") or (“compulsive eating” or “compulsive vomiting” or “compulsive purging”) OR ("bipolar disorder" or mania or manic))

and ("asylum seek*" or refugee* or migrant* or immigrant* or "forcibly displaced")

**Open Grey**

(("Depressive Disorder" OR depression OR dysthymic disorder) OR ("anxiety disorder" OR "panic disorder" OR anxiety OR agoraphobia OR separation anxiety OR neurosis OR psychoneurosis) OR ("obsessive-compulsive" OR "obsessive compulsive") OR (PTSD OR "post-traumatic stress disorder" OR "post traumatic stress disorder" OR "posttraumatic stress disorder") OR (“combat disorder” OR “neurotic disorder” OR “reactive disorder” OR “somatoform disorder” OR “somatisation disorder” OR “somatization disorder” OR “adjustment disorder” OR “dissociation disorder”) OR (schizophrenia OR psychotic OR suicidality OR "delusional disorder") OR ("substance abuse" OR "drug abuse") OR ("personality disorder" OR "avoidant personality disorder" OR "narcissistic personality disorder" OR " obsessive compulsive personality disorder" OR "anankastic personality disorder" OR psychopath OR psychopathy OR "antisocial personality disorder" OR "borderline personality disorder" OR "compulsive personality disorder" OR "dependent personality disorder" OR "histrionic personality disorder" OR "paranoid personality disorder" OR "passive-aggressive personality disorder" OR "schizoid personality disorder" OR "schizotypal personality disorder" OR psychopathic) OR ("eating disorders" OR "Anorexia Nervosa" OR "Binge-Eating Disorder" OR "binge eating disorder" OR "Bulimia Nervosa") OR (“compulsive eating” OR “compulsive vomiting” OR “compulsive purging”) OR ("bipolar disorder" OR mania OR manic))

AND ("asylum seek*" OR refugee* OR migrant* OR immigrant* OR "forcibly displaced")

**NGO and Governmental Websites Searched**: Refugee Council, Amnesty International, Human Rights Watch, Refugee Action, UNHCR, UK Home Office.

**Appendix C**

Newcastle-Ottawa Scale adaptations in Herzog et al.’s (2013) cross-sectional version: a) In Section 2 (Selection), a star was given if studies referenced a population size to justify their sample size. A power calculation was not necessary; b) In Section 3 (Selection), a star was given if non-respondents were described in any way, a summary of non-respondent categories was not necessary; c) In Section 4 (Selection) Risk factors assessed with a scale (including the PMLD) were given two stars, those assessed through single items one star - this was also applied to cohort quality assessment; d) Employment was chosen as a key confounder to control for. If studies did not control for this, the maximum they could receive in Section 1 (Comparability) was one star - this was also applied to cohort quality assessment; e) In Section 1 (Outcome), diagnostic or validated measures of mental health were given two stars - this was also applied to cohort quality assessment.

**Appendix D**

| LEAD AUTHOR | YEAR PUBLISHED | TITLE |
| --- | --- | --- |
| Alemi | 2016 | Impact of Postmigration Living Difficulties on the Mental Health of Afghan migrants Residing in Istanbul |
| Bhui | 2012 | Forced residential mobility and social support: impacts on psychiatric disorders among Somali migrants |
| Boersma | 2005 | Depression and somatization in community based asylum-seekers |
| Bogic | 2012 | Factors associated with mental disorders in long-settled war refugees: Refugees from the former Yugoslavia in Germany, Italy and the UK |
| Carswell | 2009 | The relationship between trauma, post-migration problems and the psychological well-being of refugees and asylum seekers |
| Chu | 2012 | Effects of post-migration factors on PTSD outcomes among immigrant survivors of political violence |
| Cummings | 2011 | Correlates of Depression among Older Kurdish Refugees |
| Eisen | 2016 | The Impact of Post-Migration Factors on PTSD and Depressive Symptoms Among Asylum Seekers in the United States |
| George | 2015 | Demographic characteristics, migration traumatic events and psychological distress among Sri Lankan Tamil refugees : a preliminary analysis |
| Gerritsen | 2006 | Physical and mental health of Afghan, Iranian and Somali asylum seekers and refugees living in the Netherlands |
| Groen | 2019 | Cultural Identity Confusion and Psychopathology |
| Hecker | 2018 | Differential Associations Among PTSD and Complex PTSD Symptoms and Traumatic Experiences and Postmigration Difﬁculties in a Culturally Diverse Refugee Sample |
| Heeren | 2012 | Mental health of asylum seekers: a cross-sectional study of psychiatric disorders |
| Hocking | 2015 | Mental disorders in asylum seekers: the role of the refugee determination process and employment |
| Idemudia | 2013 | Trauma exposures and posttraumatic stress among Zimbabwean refugees in South Africa |
| Jarallah | 2019 | Gender disparities and psychological distress among humanitarian migrants in Australia: a moderating role of migration pathway? |
| Kelly | 2010 | A study of migrants mental health status and engagement with adult mental health services in inner-city Dublin |
| Laban | 2005 | Postmigration living problems and common psychiatric disorders in Iraqi asylum seekers in the Netherlands |
| Maharaj | 2017 | Food insecurity and risk of depression among refugees and immigrants in South Africa |
| McColl | 2006 | Characteristics and needs of asylum seekers and refugees in contact with London community mental health teams : a descriptive investigation |
| Minihan | 2018 | Patterns and predictors of posttraumatic stress disorder in refugees: A latent class analysis |
| Molsa | 2017 | Mental and somatic health and pre- and post-migration factors among older Somali refugees in Finland |
| Morgan | 2017 | Exploring the relationship between postmigratory stressors and mental health for asylum seekers and refused asylum seekers in the UK |
| Müller | 2018 | The Negative Impact of an Uncertain Residence Status: Analysis of Migration-Related Stressors in Outpatients with Turkish Migration Background and Psychiatric Disorders in Germany Over a 10-Year Period (2005–2014) |
| Nakash | 2017 | The association between perceived social support and posttraumatic stress symptoms among Eritrean and Sudanese male asylum seekers in Israel |
| Newnham | 2019 | The mental health effects of visa insecurity for refugees and people seeking asylum: a latent class analysis |
| Nickerson | 2015 | Moral injury in traumatized refugees |
| Nose | 2018 | Prevalence and Correlates of Psychological Distress and Psychiatric Disorders in Asylum Seekers and Refugees Resettled in an Italian Catchment Area |
| Orosa | 2011 | Belief systems as coping factors in traumatized refugees: a prospective study |
| Richter | 2018 | Prevalence of psychiatric diagnoses in asylum seekers with follow-up |
| Rosner | 2019 | Prolonged grief disorder among asylum seekers in Germany: the influence of losses and residence status |
| Ryan | 2008 | Psychological distress and the asylum process: a longitudinal study of forced migrants in Ireland |
| Schock | 2015 | Impact of asylum interviews on the mental health of traumatized asylum seekers |
| Schock | 2016 | Impact of new traumatic or stressful life events on pre-existing PTSD in traumatized refugees: results of a longitudinal study |
| Silove | 2002 | Towards a researcher-advocacy model for asylum seekers: a pilot study amongst East Timorese living in Australia |
| Silove | 1997 | Anxiety, depression and PTSD in asylum-seekers : associations with pre-migration trauma and post-migration stress |
| Silove | 2007 | The impact of the refugee decision on the trajectory of PTSD, anxiety, and depressive symptoms among asylum seekers: a longitudinal study |
| Slonim-Nevo | 2015 | Risk factors associated with culture shock among asylum seekers from Darfur |
| Song | 2010 | Psychological distress in torture survivors : pre- and post-migration risk factors in a US sample |
| Steel | 1999 | Pathways from war trauma to posttraumatic stress symptoms among Tamil asylum seekers, refugees, and immigrants |
| Tinghög | 2016 | Nyanlända och asylsökande i Sverige |
| van Willigen | 1995 | Health problems of refugees in The Netherlands |
| Whitsett | 2017 | Do resettlement variables predict psychiatric treatment outcomes in a sample of asylum-seeking survivors of torture? |
| Winkler | 2018 | The Influence of Residence Status on Psychiatric Symptom Load of Asylum Seekers in Germany |
| Wong | 2016 | A lost tribe in the city: health status and needs of African asylum seekers and refugees in Hong Kong |
